# Tenascin-C predicts IVIG non-responsiveness and coronary artery lesions in kawasaki disease in a Chinese cohort

**DOI:** 10.3389/fped.2022.979026

**Published:** 2022-12-13

**Authors:** Yujie Li, Ziqing Xu, Lin Wu, Xuecun Liang, Lu Zhao, Fang Liu, Feng Wang

**Affiliations:** Department of Cardiology, Children's Hospital of Fudan University, Shanghai, China

**Keywords:** tenascin-C, IVIG non-responsiveness, coronary artery lesions, children, Kawasaki disease

## Abstract

**Objectives:**

To assess the predictive value of tenascin-C (TN-C) for intravenous immunoglobulin (IVIG) non-responsiveness and coronary artery lesions (CALs) development at the acute stage of Kawasaki disease, and to build novel scoring systems for identifying IVIG non-responsiveness and CALs.

**Methods:**

A total of 261 patients in acute-stage Kawasaki disease were included. Serum samples before IVIG initiation were collected and TN-C expression levels were measured using an enzyme-linked immunosorbent assay. In addition to TN-C, another fifteen clinical and laboratory parameters collected before treatment were compared between IVIG responsive and non-responsive groups, and between groups with and without CALs. Multiple logistic regression analyses were performed to construct new scoring systems for the prediction of IVIG non-responsiveness and CALs development.

**Results:**

IVIG non-responsive group (*n *= 51) had significantly higher TN-C level compared to IVIG responsive group (*n *= 210) (15.44 vs. 12.38 IU/L, *P* < 0.001). A novel scoring system composed of TN-C, total bilirubin, serum sodium and albumin was established to predict IVIG non-responsiveness. Patients with a total score ≥ 2 points were classified as high-risk cases. With the sensitivity of 78.4% and specificity of 73.8%, the efficiency of our scoring system for predicting IVIG non-responsiveness was comparable to the Kobayashi system. Consistently, the group developing CALs at the acute stage (*n *= 42) had significantly higher TN-C level compared to the group without CALs (*n *= 219) (19.76 vs. 12.10 IU/L, *P* < 0.001). A new scoring system showed that patients with elevated TN-C, platelet count ≥ 450 × 10^9^/L, and delayed initial infusion of IVIG had a higher risk of developing CALs. Individuals with a total score ≥ 3 points were classified as high-risk cases. The sensitivity and specificity of the novel simple system for predicting CALs development were 83.3% and 74.0%, respectively, yielding a better efficiency than the Harada score.

**Conclusion:**

Elevated TN-C appeared to be an independent risk factor for both IVIG non-responsiveness and CALs in Chinese children with KD. Our scoring systems containing TN-C is simple and efficient in the early identification of high-risk KD cases that could benefit from more individualized medications.

## Introduction

Kawasaki disease (KD), as an acute and self-limited vasculitis, is one of the leading causes of acquired heart disease in children. Nearly 15%–25% of untreated patients develop coronary artery lesions (CALs) (1), resulting in coronary artery stenosis, cardiac infarction, ventricular tachycardia, or even sudden death (2–5). Of the patients who have received the initial IVIG therapy, about 20% are intravenous immunoglobulin (IVIG) non-responders (1, 6). IVIG non-responsiveness has been suggested as an independent risk factor for CALs development (7, 8). Therefore, early prediction of IVIG non-responsiveness and CALs development at the acute stage can provide guidance for further medications in KD.

Several scoring systems have been constructed for early identification of IVIG non-responders and patients at high risk of developing CALs, such as Kobayashi (9), Egami (10), Sano (11) and Harada (12) systems. However, these predictive models were lack of consistency in clinical practice in different populations. The Japanese scoring systems showed low sensitivities and specificities in predicting IVIG non-responsiveness and CALs development in North American populations (13) and in Caucasians (14). Similarly, the Harada scoring system in American population only yielded the sensitivity and specificity of 50% and 59% in Iranian population in predicting CALs development, respectively (15). Besides, most of the available predictive models include sophisticated parameters which make it inconvenient for evaluation. Therefore, a novel biomarker or simple scoring system is needed to yield better prediction performance and to stratify high-risk KD patients.

Tenascin-C (TN-C) is one of the newly reported risk factors for IVIG non-responsiveness and CALs development. TN-C is an inflammation-related glycoprotein in the extracellular matrix, and is sparsely detected in heart tissues in healthy adults, except at the base of valve leaflets (16) and in chorda tendineae of papillary muscles (17). The expression of TN-C increased under certain pathological conditions like in dilated cardiomyopathy, myocarditis, and myocardial infarction (18–20). In addition, with the EGF-like repeat domain modulating cell adhesion and motility, TN-C serves as a regulator in the recruitment of inflammatory cells in the acute phase of cardiovascular inflammation (21–23). Compared with healthy children, TN-C levels of KD patients were found significantly elevated in a multi-center retrospective study in Japanese population, especially for the IVIG-resistant patients, showing its potential to be a novel indicator for the severity of cardiovascular inflammation in KD patients (24). However, there is no relevant data of TN-C expression during the acute phase of KD and its prediction efficacy in IVIG non-responsiveness and CALs development in Chinese population. In the present study, we reported that increased TN-C expression level in acute phase of KD predicted IVIG non-responsiveness and CALs in a Chinese cohort. Furthermore, we established simple and novel scoring systems by combining TN-C with other commonly used clinical parameters.

## Materials and methods

### Subjects

We conducted a retrospective and single-centered study in Children's Hospital of Fudan University. Two hundred and sixty-one hospitalized KD children were enrolled from October 2018 to November 2021. According to the AHA guidance published in 2017 (1), the diagnostic criteria for complete KD (CKD) include fever for at least 4 days associated with ≥ 4 of the following 5 main features, including (1) conjunctivitis without exudates; (2) changes in extremities; (3) cervical lymphadenopathy; (4) rash; and (5) oral mucosa lesions. And incomplete KD (IKD) is defined as fever ≥ 5 days and 2 or 3 compatible main features and accompanied by positive laboratory tests or echocardiogram. Patients diagnosed with both CKD and IKD were included. And the following cases were excluded: (1) patients who had underlying cardiovascular conditions, such as congenital heart disease, cardiomyopathy, and arrhythmia; (2) patients who suffered from autoimmune disease; (3) recurrent KD patients; (4) patients who refused IVIG treatment; (5) patients who had received IVIG infusion before admission; (6) patients with incomplete data. During the same period, 30 sex- and age-matched healthy controls who underwent routine evaluation in child health department were also enrolled as the control group. The study was approved by the Ethics Committee of Children's Hospital of Fudan University (2018–165) and written informed consent was obtained from each patient's guardian.

IVIG non-responsiveness is defined as persistent or recrudescent temperature of more than 38°C between 36 h and less than 7 days after the completion of initial IVIG infusion (1). Coronary artery involvement was assessed by echocardiography before the initial treatment and followed once or twice a week during the acute stage. CALs was confirmed with the *Z* score ≥2.5 based on the database created by Children's Hospital of Boston (http://zscore.chboston. org/), including the left main coronary artery, left anterior descending coronary artery, left circumflex branch, and proximal and middle segments of right coronary artery.

According to the responsiveness to the initial IVIG infusion, the patients were classified into those who responded to (IVIG+) or resisted the initial IVIG infusion (IVIG-); similarly, those who developed CALs (CALs) or did not develop CALs (NCALs) were categorized in all KD cases according to *Z* scores of coronary arteries.

### Treatment strategy of KD in acute stage

All the KD patients received a standard single infusion of IVIG (2 g/kg) over 10–12 h once the diagnosis was established, together with high-dose Aspirin (30–50 mg/kg/day). Low-dose Aspirin of 3–5 mg/kg/day was given after cessation of fever for 3 days and C-reactive protein returned to normal values (1, 6). Notably, no extra interventions were conducted according to the prediction outcomes of our TN-C scoring systems in KD children.

### Blood samples collection and serum TN-C levels measurement

Blood samples of KD patients were collected after laboratory detection and before the administration of initial IVIG, respectively. Serum was separated by centrifugation at 3000 r/min for 15 min at 4°C. Samples were then stored at −70°C until analyzed. The frozen samples were tested once 30 subjects were enrolled, and added into 96-well plate in triple replication until detected. Serum TN-C levels were measured by enzyme-linked immunosorbent assay kit (Keshun Science and Technology Corporation, Shanghai, China) according to the manufacturer's instructions.

### Data collection

Data extracted from electronic medical records include the diagnosis of CKD or IKD, demographic data (age and sex), the responsiveness to IVIG treatment, CALs, febrile days at the initial IVIG infusion, and chemistry panels [white blood cell (WBC) count, platelet count (PLT), hemoglobin (HB), alanine aminotransferase (ALT), aspartate transaminase (AST), total bilirubin (TB), albumin (ALB), hematocrit (Hct), CRP, neutrophil percentage (NEU) and serum sodium level].

### Statistical methods

Data were analyzed by SPSS software version 26.0. Numerical data were presented as mean ± SD and median combined with interquartile range for data of normal distribution and non-normal distribution, respectively. One-sample Kolmogorov-Smirnov Test was applied to determine whether variables follow a normal distribution. Comparisons between two groups were conducted by student's *t*-test or Mann-Whitney *U* test for normally and non-normally distributed data, respectively. Categorical variables were presented as percentage and compared by Chi-square test.

Binary logistic regression analyses were performed to identify the independent risk factors for IVIG non-responsiveness and CALs. To determine the cut-off values of each independent risk factor, receiver-operating characteristic (ROC) curves were plotted, and the maximal Youden indexes were calculated. According to each regression coefficient, a set of scores were assigned for the independent risk factors to establish our scoring systems. To assess the sensitivity and specificity of the novel scoring systems, the ROC curves were plotted and the areas under the curve (AUC) were calculated. A two-tailed *P* value less than 0.05 was considered statistically significant.

## Results

### Demographics and clinical characteristics

Among the 261 enrolled KD patients, 8.4% (*n *= 22) children were younger than 6 months and 12.7% (*n *= 33) older than 5 years, with the age range of 14 days to 10 years. The ratio of male to female was 1.42:1. Of the 261 cases, 33 (12.6%) were diagnosed as IKD. A total of 185 (70.9%) children received IVIG infusion between 5 and 10 days after the onset of fever, 71 patients (27.2%) at the 4th day, and the other 5 (1.9%) after 10 days. Fifty-one (19.5%) patients showed IVIG non-responsiveness, and 42 (16.1%) developed CALs. No significant difference was observed in terms of age and sex either between IVIG + and IVIG- groups, or the CALs and NCALs groups. There was no significant difference in percentage of IVIG non-responsiveness between CKD (46/228) and IKD (5/33) groups (20.2% vs. 15.2%, *P *> 0.05), while the ratio of CALs in IKD (10/33) was significantly higher than that of CKD (32/228) (30.3% vs. 14.0%, *P* < 0.05).

### TN-C levels were significantly elevated in KD patients at the acute phase

Serum TN-C levels of KD patients at the acute phase were significantly elevated compared with those of healthy controls [13.05 (9.66,16.79) vs. 10.14 (6.98,13.09) IU/L, *P *< 0.001]. TN-C levels of IVIG- group were significantly higher than those of IVIG + group [15.44 (11.81, 19.18) vs. 12.38 (9.14, 15.95) IU/L, *P *< 0.001]. CALs patients showed much higher TN-C levels than NCALs patients [19.76 (14.79, 23.22) vs. 12.10 (9.35, 15.11) IU/L, *P *< 0.001]. No statistical difference of TN-C level was observed between CKD and IKD patients [12.91 (9.64, 16.78) vs. 13.46 (10.66, 16.79) IU/L, *P *> 0.05].

### TN-C predicts IVIG non-responsiveness and CALs development

According to the ROC curve analysis of TN-C, a cut-off value of 13.47 IU/L yielded a sensitivity and specificity of 68.6% and 59.0%, respectively, in identifying IVIG non-responsiveness, with an AUC of 0.683 (95% CI 0.608∼0.724). Meanwhile, a cut-off value of 16.86 IU/L yielded a sensitivity of 72.5%, and a specificity of 84.3% in predicting CALs development, with an AUC of 0.856 (95% CI 0.73–0.920).

### Scoring system efficacy in IVIG resistance prediction

**Figure 1:**
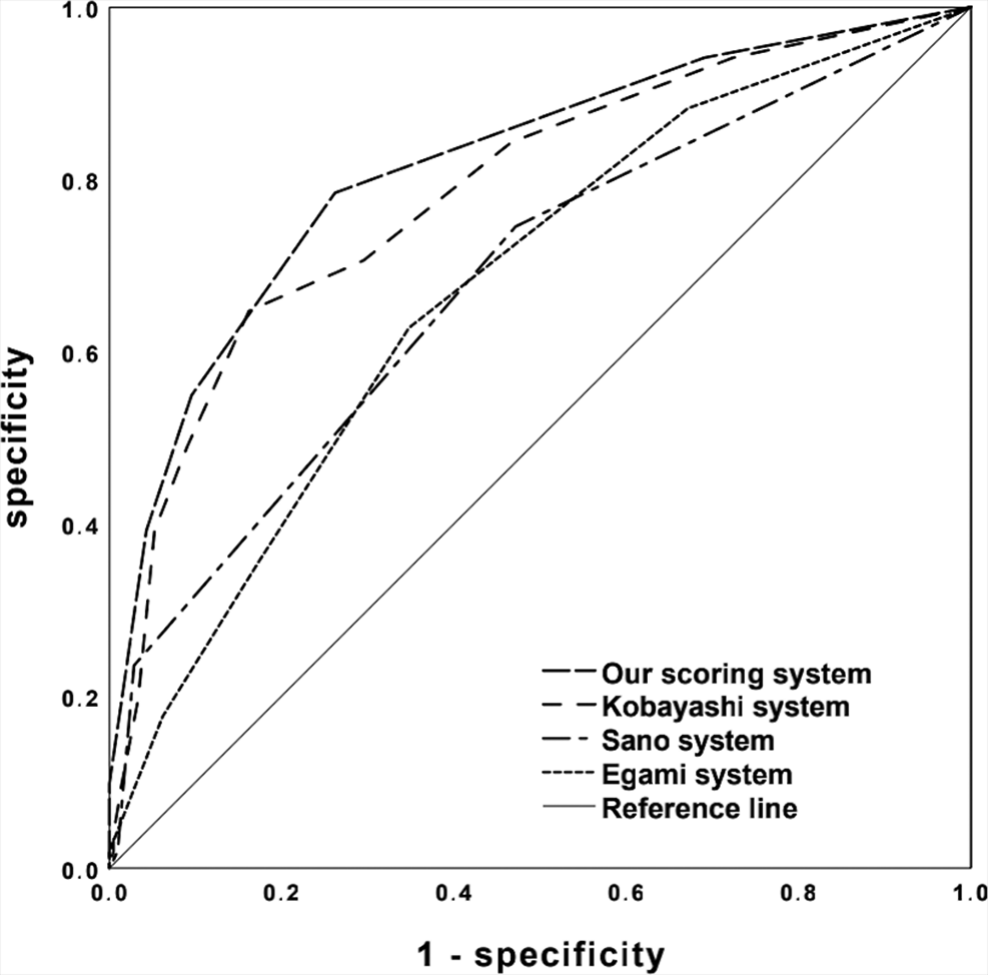
ROC curves of the new simple scoring model, Kobayashi, Egami, and Sano scoring systems in predicting IVIG non-responsiveness.

Univariable analysis revealed significant differences between IVIG+ and IVIG- group in terms of 10 factors including TN-C (Table 1). All the 10 variables were included in the following stepwise backward logistic regression analysis. Results suggested that elevated TN-C and TB, low ALB and sodium were independent risk factors for IVIG non-responsiveness. To determine the cut-off value of each risk factor, ROC curves were plotted and Youden indexes were calculated. According to the regression coefficient, our scoring system for predicting IVIG non-responsiveness was established including TN-C ≥ 13.47 IU/L, TB ≥ 7.45 μmol/l, ALB ≤ 32.3 g/L, and sodium ≤ 133 mmol/L (Table 2). The Hosmer-Lemeshow statistic was 0.596 (*P *> 0.05), achieving a good fit with the regression model. Based on a total score of 5 points, a cut-off value of 2 points yielded a sensitivity of 78.4% and specificity of 73.8%, respectively, in predicting IVIG non-responsiveness. And the AUC for this novel scoring system was 0.816 (95% CI 0.747–0.886) (Figure 1). Kobayashi (9), Egami (10) and Sano (11) systems yielded the sensitivities and specificities of 64.7% and 83.8%, 35.3% and 82.9%, and 23.5% and 97.1%, respectively. In contrast, the scoring systems in Chinese population, i.e., Yang (25), Xiao (26), and Xie (27) systems, yielded sensitivities and specificities of 76.5% and 62.4%, 62.7% and 81.0%, and 72.5% and 65.7%, respectively, in verification in our cohort (Supplementary Figure). Comparatively, our scoring system suggested a higher sensitivity in predicting IVIG non-responsiveness (Figure 1).

**Table 1:** Comparison of clinical and laboratory characteristics between the IVIG + and IVIG- KD patients.

	IVIG + (*n *= 210)	IVIG- (*n *= 51)	*P* value
Male (*n*, %)	124 (59.0%)	31 (60.7%)	0.977
Age (months)	22.0 (13.0, 36.3)	28.0 (16.0, 52.0)	0.077
Days at the initial IVIG infusion (days)	5.0 (5.0, 6.0)	5.0 (4.0, 6.0)	0.011
TN-C (IU/L)	12.38 (9.14, 15.95)	15.44 (11.81, 19.18)	<0.001
ALT (IU/L)	23.00 (14.85, 51.75)	42.00 (23.80, 135.20)	0.01
AST (IU/L)	28.55 (23.60, 38.50)	36.00 (26.80, 67.90)	0.01
TB (*μ*mol/L)	5.35 (4.10, 8.45)	8.50 (5.20, 19.20)	<0.001
ALB (g/L)	35.25 (32.88, 38.30)	33.80 (31.20, 36.20)	0.002
Sodium (mmol/L)	136 (135, 138)	133 (131, 135)	<0.001
Hb (g/L)	111 (103, 118)	112 (105, 118)	0.969
Hct (%)	33.26 (30.975, 35.25)	33.40 (31.80, 35.20)	0.850
WBC (×10^9^/L)	12.95 (10.28, 16.33)	14.10 (11.40, 17.00)	<0.001
NEU (%)	66.45 (51.50, 75.85)	79.20 (70.00, 86.50)	0.141
PLT (×10^9^/L)	353 (275, 453)	297 (260, 368)	0.01
CRP (mg/L)	61.00 (36.74, 94.55)	89.00 (57.00, 148.00)	<0.001

**Table 2:** Logistics regression analysis and the simple scoring system for predicting IVIG non-responsiveness.

Variables	*β*	SE	OR (95% CI)	*P* value	Score
TN-C ≥ 13.47 IU/L	1.173	0.384	3.233 (1.524∼6.858)	0.002	1
ALB ≤ 32.3 g/L	0.973	0.410	2.647 (1.185∼5.910)	0.018	1
Sodium ≤ 133 mmol/L	1.932	0.392	6.900 (3.202∼14.869)	<0.001	2
TB ≥ 7.45 μmol/L	1.137	0.386	3.116 (1.462∼6.642)	0.003	1

### Scoring system efficacy in CALs development prediction

**Figure 2:**
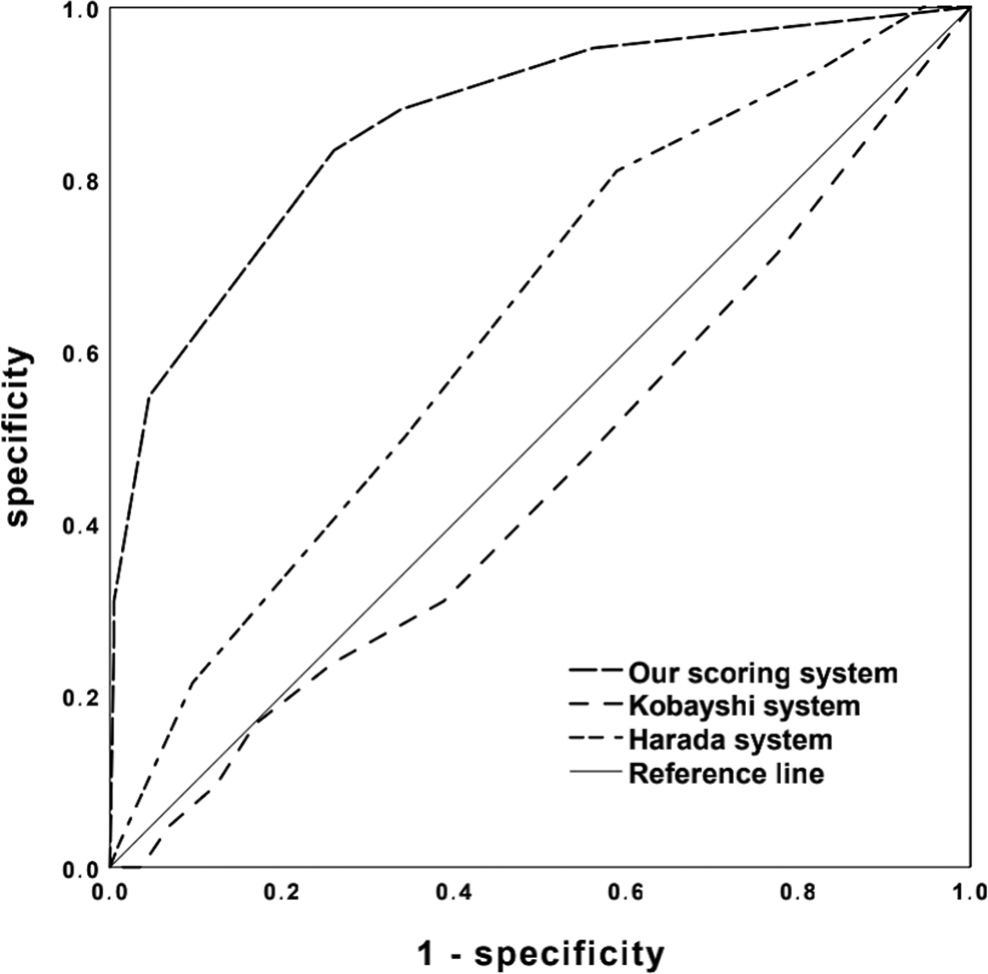
ROC curves of the novel simple scoring model, Kobayashi and Harada scoring systems in predicting CALs development.

Seven factors showed significant differences between NCALs and CALs groups (Table 3). The next stepwise backward logistic regression analysis suggested that elevated TN-C and PLT, together with delayed infusion of IVIG, significantly increased the risk of developing CALs. According to the ROC curves and the maximal Youden index, the novel scoring system with a total score of 6 points was established including TN-C ≥ 16.86 IU/L, PLT ≥ 450 × 10^9^/L, and illness course at the initial IVIG infusion ≥ 6 days (Table 4). A cut-off value of 3 points achieved a sensitivity of 83.3% and specificity of 74.0%, respectively, in predicting CALs. And the AUC for this novel scoring system was 0.866 (95% CI 0.803–0.929) (Figure 2). Compared with our novel scoring system for predicting CALs, Harada (12) system showed a lower sensitivity and specificity of 81.0% and 41.1%, respectively, while the AUC of Kobayashi (9) score was even less than 0.5.

**Table 3:** Comparison of clinical and laboratory characteristics between patients with CALs and NCALs.

	NCALs (*n *= 219)	CALs (*n *= 42)	*P* value
Male (*n*, %)	125 (57.0%)	30 (71.4%)	0.083
Age (months)	23.0 (14.0, 39.0)	19 (10.3, 35.0)	0.142
Days at the initial IVIG infusion (days)	5.0 (4.0, 6.0)	6.0 (5.0, 7.0)	0.002
IKD	23 (69.7%)	10 (30.3%)	0.017
TN-C (IU/L)	12.10 (9.35, 15.11)	19.76 (14.79, 23.22)	<0.001
ALT (IU/L)	27.00 (16.00, 74.00)	23.95 (13.03, 47.98)	0.248
AST (IU/L)	29.20 (24.00, 43.00)	26.95 (21.75, 38.80)	0.096
TB (μmol/L)	6.00 (4.30, 9.20)	4.85 (3.30, 8.70)	0.066
ALB (g/L)	35.40 (32.70, 38.20)	34.05 (31.68, 35.55)	0.005
Sodium (mmol/L)	136 (134, 137)	135.5 (132.75, 137)	0.294
CRP (mg/L)	68.00 (37.00, 103.00)	65.00 (44.48, 123.50)	0.368
Hb (g/L)	111 (105, 119)	105 (100, 114)	0.01
Hct (%)	33.52 (31.40, 35.52)	31.70 (29.42, 33.63)	0.002
WBC (×10^9^/L)	12.8 (10.20, 16.30)	14.2 (11.98, 17.83)	0.037
NEU (%)	69.80 (56.50, 78.20)	66.55 (46.75, 76.48)	0.280
PLT (×10^9^/L)	324 (263, 421)	420 (314, 506)	0.001

**Table 4:** Logistics regression analysis and the simple scoring system for predicting CALs.

Variables	*β*	SE	OR (95% CI)	*P* value	Score
TN-C ≥ 16.86 IU/L	3.134	0.492	22.955 (8.748∼60.235)	<0.001	3
Days at the initial IVIG infusion ≥6 days	1.073	0.442	2.923 (1.228∼6.956)	<0.001	1
PLT ≥ 450 × 10^9^/L	1.847	0.571	6.342 (2.303∼17.462)	0.015	2

## Discussion

Timely infusion of the initial IVIG in KD could decrease CALs development, and some risk factors for IVIG non-responsiveness are also believed to increase the susceptibility to develop CALs (28, 29). Combinations of initial IVIG therapy and additional medications, such as glucocorticoid, ciclosporin, ulinastatin and infliximab, have been reported to be effective in attenuating the inflammatory cytokines, shortening the duration of fever, and decreasing the incidence of CALs in high-risk patients (30–36). Therefore, accurate predictions of IVIG non-responsiveness and CALs are of great significance in making individualized treatment strategy according to the patients' risk stratifications, which may avoid abuse of immunosuppressors in those with low risks and reduce the severe side effects and the financial cost.

Considering the insufficient efficacy of the available scoring systems in identification of IVIG non-responders in different populations, recent studies have focused on seeking novel reliable indicators to stratify high-risk KD children. Among them, TN-C is believed to be a promising biomarker for the severity of acute inflammation and a regulator for cell behaviors such as proliferation, differentiation, and migration, as well as tissue healing and regeneration (37). For KD patients in Japan population, the cut-off value of 95.2 ng/ml of TN-C yielded a sensitivity of 58% and specificity of 78% to predict IVIG resistance (24). Similarly, our study demonstrated that TN-C was significantly elevated in IVIG non-responsive patients, and TN-C as a single indicator yielded a comparable sensitivity of 68.6% in Chinese patients with a cut-off value of 13.47 IU/L. In addition, our simple scoring system showed the highest sensitivity and maximal Youden index among the 7 scoring systems in predicting IVIG non-responsiveness in the present study. Comparatively, scoring systems in Japanese patients showed significantly lower sensitivities than those in Chinese in our cohort. Especially, the classic Sano scoring system yielded the lowest sensitivity of 23.5%. Furthermore, our novel scoring system contains only 4 variables, which might be helpful to simplify the workflow of predicting IVIG non-responsiveness.

Although the incidence of CALs has been reduced significantly after the initiation of IVIG, KD patients with severe CALs involvement at the acute stage are still at the residual risk of myocardial ischemia resulting from coronary artery thrombosis and stenosis (1). Therefore, accurate prediction of CALs is necessary for timely application of additional medications. However, the current scoring systems are not efficient in prediction of CALs development in different populations. For example, the Formosa scoring system only yielded a sensitivity of 33% to predict CALs when validated in a Chinese cohort from Anhui province (38, 39). And in consideration of the low specificities of Liu (39) and Hua (40) scoring systems of 41.8% and 51.4%, respectively, the currently available Chinese population-based scoring systems were not ideal for predicting CALs.

During the acute stage of KD, TN-C expression in coronary arteries and myocardial tissues was reported to coincide with the areas where inflammatory cells infiltrated, and its intensity reflected the extent of inflammation (41). While in the remote-stage cases, only weak expression of TN-C was observed in the luminal surface of dilated coronary aneurysms and in the thickened intima of the recanalized vessels after thrombotic occlusion (41). Okuma et al. (24) found that the acute-stage KD patients with CALs showed significantly higher TN-C level than those without CALs (139.3 ng/ml vs. 70.3 ng/ml, *P* = 0.001). Furthermore, TN-C expression was significantly higher in initial therapy non-responders than in the responders (42). Consistently, our study revealed that the expression of TN-C was significantly higher in CALs children in the acute phase. With a cut-off value of 16.86 IU/L, TN-C yielded a sensitivity of 72.5% and a specificity of 84.3% in predicting CALs development, suggesting that TN-C itself might be a nice indicator for predicting CALs. In combination with PLT and the illness course of IVIG infusion, our scoring system showed better predictive efficiency than Harada score, with sensitivities of 83.3% and 81.0%, and specificities of 74.0% and 41.1%, respectively. In contrast, Kobayashi system was invalid in identifying CALs in our Chinese cohort with the AUC below 0.5. Notably, previous studies reported that the infusion of IVIG after 10 days of the illness course was an independent risk factor for CALs (43, 44), yet our corresponsive data was 6 days. The reasons might be due to renewal of the diagnostic criteria and the suggestion of IVIG infusion as soon as the diagnosis can be established (1).

However, the present study has some limitations. First, it is retrospective and single-centered. Second, large sample-sized studies are in need to validate the role of TN-C in KD patients of different populations. Besides, indicators like N-terminal pro-brain natriuretic peptide, calcitonin, weight for height, D-dimer, eosionphils, interleukin-6, interleukin-10, and ferritin were not included in the present study (29, 45, 46). Given the absence of some clinical parameters, the effectiveness of part of reported systems to predict CALs in Chinese population has not been validated in our study. Finally, febrile controls are absent in our study. As a non-specific indicator, TN-C was reported to be elevated in other inflammatory diseases in children, including COVID 19 infection, sepsis, mycobacterium tuberculosis infection, and methicillin-resistant staphylococcus aureus-induced pneumonia (47–50). Since the diagnosis of KD is based on exclusion of other clinically similar entities, further studies are required to investigate the differences of TN-C expression between KD and other inflammatory diseases.

## Conclusion

TN-C appeared to be an independent risk factor for IVIG non-responsiveness and CALs development in Chinese children with KD. The proposed scoring system, that incudes this protein, is simple and efficient in the early identification of high-risk KD patients that could benefit from timely individualized treatment strategies.

## Data Availability

The raw data supporting the conclusions of this article will be made available by the authors, without undue reservation.
