# Supplementary figures and images for: Tenascin-C predicts IVIG non-responsiveness and coronary artery lesions in kawasaki disease in a Chinese cohort
Source: Front Pediatr. 2022 Dec 13;10:979026. doi: 10.3389/fped.2022.979026 (PMC9792982; doi:10.3389/fped.2022.979026)

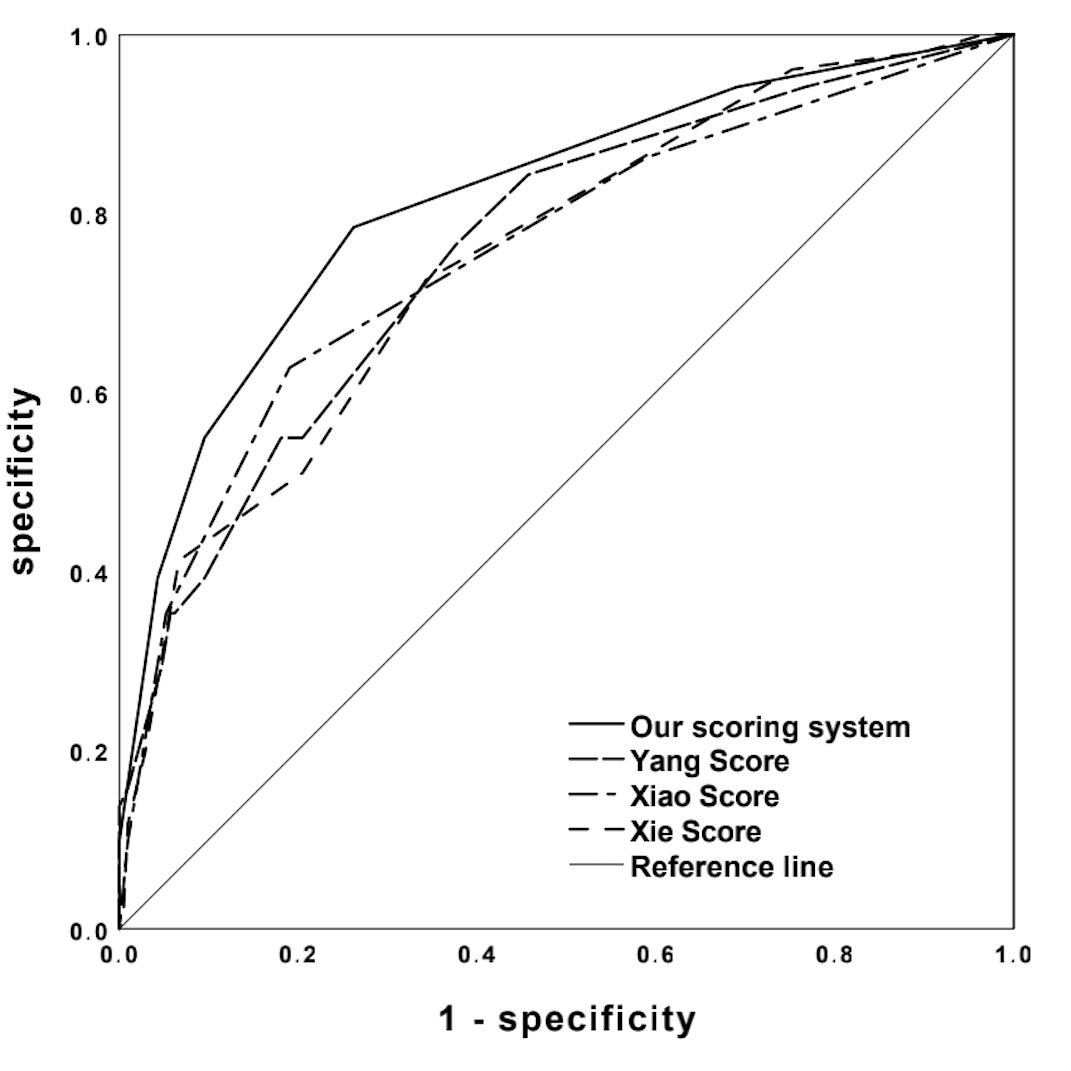

Supplement: Supplementary file 1 [file Image1.jpeg]
